# Genetic and phenotypic characterization of the heat shock response in *Pseudomonas putida*

**DOI:** 10.1002/mbo3.217

**Published:** 2014-10-10

**Authors:** Fumihiro Ito, Takayuki Tamiya, Iwao Ohtsu, Makoto Fujimura, Fumiyasu Fukumori

**Affiliations:** 1 Graduate School of Life Sciences, Toyo UniversityGunma; 2 Faculty of Life Sciences, Toyo UniversityGunma; 3 Graduate School of Biological Sciences, Nara Institute of Science and TechnologyNara, Japan; 4 Faculty of Food and Life Sciences, Toyo UniversityGunma

**Keywords:** Molecular chaperone, protein disaggregation, *Pseudomonas putida*

## Abstract

Molecular chaperones function in various important physiological processes. Null mutants of genes for the molecular chaperone ClpB (Hsp104), and those that encode J-domain proteins (DnaJ, CbpA, and DjlA), which may act as Hsp40 co-chaperones of DnaK (Hsp70), were constructed from *Pseudomonas putida* KT2442 (KT) to elucidate their roles. The KTΔ*clpB* mutant showed the same heat shock response (HSR) as the wild-type, both in terms of heat-shock protein (Hsp) synthesis (other than ClpB) and in *hsp* gene expression; however, the mutant was quite sensitive to high temperatures and was unable to disaggregate into thermo-mediated protein aggregates, indicating that ClpB is important for cell survival after heat stress and essential for solubilization of protein aggregates. On the other hand, the KTΔ*dnaJ* mutant was temperature-sensitive, and formed more protein aggregates (especially of high molecular weight) upon heat stress than did KT. *P. putida* CbpA, a probable Hsp, partially substituted the functions of DnaJ in cell growth and solubilization of thermo-mediated protein aggregates, and might be involved in the HSR which was regulated by a fine-tuning system(s) that could sense subtle changes in the ambient temperature and control the levels of *σ*^32^ activity and quantity, as well as the mRNA levels of *hsp* genes.

## Introduction

Molecular chaperones have essential roles assisting proteins in their folding and assembly, in preventing their misfolding and aggregation, and in their transport (Hartl et al. 2011). The heat shock protein (Hsp) of the 70 kDa family (Hsp70) of molecular chaperones is a major component of the cellular chaperone network and of the stress response (Genevaux et al. 2007; Hartl et al. 2011). *Escherichia coli* genome encodes three Hsp70 members; namely, DnaK, HscA, and HscC (Hennessy et al. 2005). *E*. *coli* DnaK is the most extensively characterized Hsp70 family member, and its function is important for bacterial viability. Mutations in the *dnaK* gene result in temperature sensitive phenotypes (Georgopoulos 1977; Paek and Walker 1987). *E*. *coli* DnaK consists of a highly conserved N-terminal ATPase domain of 44 kDa, and a C-terminal domain, which is divided into a conserved substrate-binding domain of 15 kDa and an immediate COOH-terminal, 10 KDa *α*-helical domain. The chaperone function of Hsp70 is regulated by the state of the bound nucleotide. When ATP is bound, the association and dissociation of client peptides to or from the substrate-binding domain occur at high rates. ATP hydrolysis, which is stimulated by the Hsp40 co-chaperone and substrate binding, results in a much slower exchange of the substrate, thereby exerting its chaperone activity in vitro and in vivo (Genevaux et al. 2007). The ATP-dependent cycle of DnaK is regulated primarily by the Hsp40 protein DnaJ and the nucleotide exchange factor GrpE (Straus et al. 1990; Szabo et al. 1994; McCarty et al. 1995; Wawrzynów et al. 1995; Laufen et al. 1999; Siegenthaler and Christen 2006). Among several Hsp40 proteins found in *E. coli*, DnaJ was shown to be the key regulator of DnaK (Hennessy et al. 2005). DnaJ is composed of four domains: a J-domain, a Gly/Phe-rich domain, a repeat of four cysteine residues that form two zinc-binding centers, and an uncharacterized C-terminal domain (Hennessy et al. 2005). In general, a J-domain assists in the interaction with an Hsp70 protein and stimulates the ATPase activity of the specific partner(s) (Hennessy et al. 2005). DnaJ binds to the hydrophobic core of peptide segments (approx. eight residues) enriched with aromatic and large aliphatic residues (Rüdiger et al. 2001), and functions in presenting non-native substrate proteins to DnaK. Depletion of DnaJ also results in temperature sensitive phenotype in *E*. *coli* (Sell et al. 1990). Two other Hsp40 members, CbpA (Ueguchi et al. 1995) and DjlA (Genevaux et al. 2001), are known to function with DnaK, in addition to DnaJ. The Gly/Phe-rich domain (Perales-Calvo et al. 2010) and one of the zinc-binding sites (Linke et al. 2003) were found to be involved in substrate binding in DnaJ. As CbpA does not contain cysteine residues, it does not have zinc-binding sites; however, CbpA shows overlapping functions with DnaJ in the chaperone activity (Genest et al. 2011), in sustaining cell growth at high temperatures (Ueguchi et al. 1995), and in the activity and stability controls of *σ*^32^ (Tatsuta et al. 1998).

**Figure 1:**
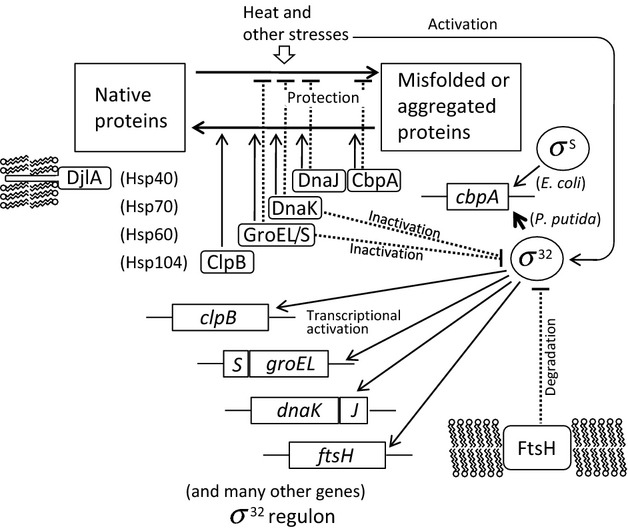
The proposed model for the heat shock response in *Escherichia coli* and *Pseudomonas putida*. The DnaK/DnaJ and GroEL/GroES molecular chaperones bind to and inactivate the heat-shock *σ* factor *σ*^32^, and the membrane-bound ATP-dependent protease degrades *σ*^32^ with assistance from these chaperones; therefore, *σ*^32^-dependent transcription is limited under normal conditions. Heat and other stresses may cause denaturation of native proteins, in which case such molecular chaperones function in protective roles. Non-native proteins titrate these chaperones from *σ*^32^, and activate the transcription of the so-called heat-shock protein genes, including molecular chaperones. The ClpB molecular chaperone and the DnaK system play important roles in the disaggregation of protein aggregates. The functions of DnaJ co-chaperone can be partially compensated by CbpA co-chaperone, for which the expression is controlled by the stationary phase-specific *σ* factor *σ*^S^ in *E*. *coli* and by *σ*^32^ in *P*. *putida*. *J*, *dnaJ*; S, GroES; *S*, *groES*.

The heat shock response (HSR) is a universal cellular response against damage to protein folding under heat and other stresses (Fig. 1). In many proteobacteria, the HSR is controlled by an alternative sigma factor, *σ*^32^, which directs RNA polymerase to the promoters of *hsp* genes (Straus et al. 1987; Morita et al. 2000; Guisbert et al. 2008). The *E. coli σ*^32^ regulon consists of about 50 transcriptional units and comprises approximately 90 genes (Nonaka et al. 2006). The regulon encodes many global transcriptional regulators and proteins that contribute to the maintenance of DNA and RNA integrity, along with canonical Hsps. The transient induction of *σ*^32^ upon heat shock, peaking at 5−15 min, is conducted by its temporal stabilization and translational upregulation (Guisbert et al. 2008). In the absence of stressors or when cells have adapted to stress conditions (shut-off stage), the level of *E*. *coli σ*^32^ declines through degradation by a membrane-bound ATP-dependent protease, FtsH (Herman et al. 1995; Tomoyasu et al. 1995; Tatsuta et al. 1998). Two major chaperone systems, DnaK/DnaJ/GrpE and GroEL (Hsp60)/GroES (Hsp10) are known to control the activity and quantity of *σ*^32^, thereby controlling the HSR via negative feedback loops (Guisbert et al. 2008). DnaK and GroEL are major ubiquitous chaperones that play crucial roles in promoting protein folding, not only under stress conditions but also during normal growth (Hartl et al. 2011). The DnaK system also functions in the disaggregation of thermo-mediated protein aggregates, in cooperation with ClpB (Hsp104) (Parsell et al. 1994; Glover and Lindquist 1998; Mogk et al. 1999; Tomoyasu et al. 2001; Doyle and Wickner 2009; Tyedmers et al. 2010; Seyffer et al. 2012). Protein disaggregation is considered to be initiated by the association of DnaK/DnaJ with the aggregate (Weibezahn et al. 2004), which allows binding of ClpB to the aggregate (Acebrón et al. 2009) and threading activity to be exerted (Schlieker et al. 2004). *E. coli* ClpB also functions in protecting cells from lethal effects of very high temperatures (Squires et al. 1991).

**Figure 2:**
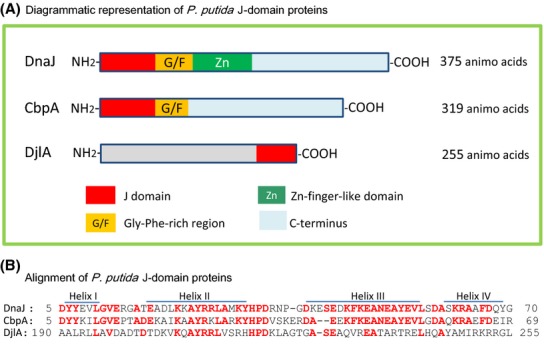
*Pseudomonas putida* J-domain proteins. (A) Diagrammatic representation of *P. putida* J-domain proteins. (B) Alignment of the J-domain of *P. putida* J-domain proteins. Identical amino acids are highlighted in red.

*Pseudomonas putida* is a ubiquitous Gram-negative bacterium that is metabolically versatile and can adapt to various environmental conditions (Timmis 2002). The strain has a relatively high intrinsic resistance to organic solvents, and a number of factors are known to be involved in the resistance ability (Ramos et al. 2002). In a previous work, we described the toluene-resistant strain of *P. putida* KT2442 (KT) that accumulated several Hsps under non-stress conditions. A point mutation in *dnaK* was shown to be the cause of the characteristic phenotypes; namely, toluene resistance, temperature sensitivity, and Hsp accumulation (Kobayashi et al. 2011). Meanwhile, a *dnaK* insertion mutant isolated from another *P. putida* strain showed growth retardation at 35°C (Dubern et al. 2005). Although the bacterial HSR has been thoroughly investigated in *E. coli*; there are not many reports that systematically describe the HSR in the genus *Pseudomonas* (Allan et al. 1988; Keith et al. 1999; Zhao et al. 2007). Since *E. coli* and *P. putida* show notable differences in the control mechanisms for a minor sigma factor, *σ*^S^ (Venturi 2003), the HSR mediated by the heat shock minor sigma factor, *σ*^32^, which is encoded by the *rpoH* gene, in *P. putida* may be different than that in *E. coli*. Three J-domain family proteins (i.e., DnaJ, CbpA, and DjlA, encoded by PP4726, PP4848, and PP0407, respectively), each of which may act as a co-chaperone of DnaK, have already been annotated in the genome of *P. putida* KT2440 (Fig. 2). Our data suggest that the HSR in *P. putida* is controlled by a system quite similar to that in *E. coli*; however, curved-DNA-binding protein CbpA, which is controlled by *σ*^S^ in *E. coli*, is a *σ*^32^-dependent Hsp in *P. putida*, and hence acts as a replacement when DnaJ is depleted. Additionally, our data indicate that posttranscriptional controls of *clpB*, *dnaK*, and *htpG* can be distinct from that of *groEL* in the strain.

## Experimental Procedures

### Bacterial strains and growth conditions

The bacterial strains used in this study are listed in Table S4. *P. putida* strain KT (Franklin et al. 1981) and its derivative strains were grown at 30°C unless otherwise stated, and *E*. *coli* strains at 37°C in LB (Lennox) medium (10 g tryptone, 5 g yeast extract, and 5 g NaCl L^−1^). Solid medium contained 1.5% (w/v) agar. A 1:500 dilution of each overnight culture was prepared with 50 mL of fresh medium, and these cultures were grown aerobically in 200 mL baffled-flasks with 200 rpm. Doubling time was determined by measuring the increase of optical density (OD_600_) while cells were in logarithmic phase. The final concentrations (in mg L^−1^) of antibiotics, when added, were as follows: ampicillin, 100; rifampicin, 100; kanamycin, 50; streptomycin, 100 (*E*. *coli*) and 250 (*P*. *putida*).

### Thermal treatment

To assess thermal sensitivity of logarithmically growing cells, 0.5 mL of the culture was transferred to a 16 mm*φ* glass tube when its OD_600_ was ˜1.0, and placed water bath at appropriate temperatures for the duration required. For stationary phase cells, overnight-grown cells equivalent to OD_600_ of 0.6 unit were mixed with LB in a total volume of 2 mL and incubated at 30°C for five doubling times of the tested strain, to reach the stationary phase. Then, 0.5 mL of the culture was transferred to a 16-mm*φ* glass tube and incubated for 5 min at 50°C. Viable counts were measured after plating serial dilutions of the culture onto the LB solid medium.

### DNA manipulations and plasmid construction

General DNA manipulations were performed as described in a published protocol (Ausubel et al. 1992). Plasmids and primers used are listed in Tables S4 and S5, respectively.

Plasmids for complementation studies were constructed as follows. DNA fragments that contain *cbpA*, *clpB*, and *dnaJ* were PCR-amplified with primers cbpA F–160Xho and cbpA R+1160Xho, primers clpB F–199Xho and clpB R+2757Xho, and primers dnaJ F–157Xho and dnaJ R+1347H, respectively. DNA fragments were digested with XhoI (*cbpA*), XhoI (*clpB*), and XhoI and HindIII (*dnaJ*), respectively, and separately cloned into the corresponding sites of pKT231 (Bagdasarian et al. 1981) to yield pKT231-cbpA, pKT231-clpB, and pKT231-dnaJ, respectively. The DNA fragments that contain *djlA* were PCR-amplified with primers djlA F–6 and djlA R+898H, ligated with a DNA fragment containing a probable cbpA promoter, which had been PCR-amplified with primers cbpA F–160Xho and cbpA R–7, digested with XhoI and HindIII and then cloned into the corresponding sites of pKT231 to yield pKT231-djlA.

### Construction of null-mutants of *P. putida* strains

The disruption of *P*. *putida* genes were carried out by means of a suicide vector pKNG101 (Kaniga et al. 1991). In order to construct a *clpB* deletion mutant, DNA fragments containing partial *P*. *putida* KT *clpB* (+489 to +1557, of which +1 represents base A of the initiation codon) were amplified by PCR with primers clpB F+489S and clpB R+1557S, and then cloned into pNEB193. Thus, the obtained recombinant plasmid was treated with SacII (located at +756 and +915), ligated with a DNA fragment containing the kanamycin-resistant gene (Km^R^) from Tn903. Subsequently, a 2.2-kb SalI DNA fragment containing a portion of *clpB*, which had been disrupted by Km^R^, was cloned into pKNG101. For disruption of *algU*, *cbpA*, *dnaJ*, and *djlA*, portions of relevant genes were similarly pCR amplified, cloned into pNEB193, treated with an appropriate restriction enzyme(s), Km^R^ cassette is inserted, and cloned into pKNG101. PCR primers, restriction enzymes used for the Km^R^ cassette insertion and the cloning step are as follows: *algU*, algU F−38B and algU R+529Xba, XhoI (located at +346), and BamHI and XbaI; *cbpA*, cbpA F–19B and cbpA R+686B, SacII (located at +264), and BamHI; *dnaJ*, dnaJ F+47B and dnaJ R+824B, NcoI (located at +567 and +609), and BamHI; *djlA*, djlA F–5S and djlA R+551B, MscI (located at +202), and SalI and BamHI. Plasmids thus constructed were introduced into *P*. *putida* strains KT or KT-R2 by electroporation. Sm-resistant transconjugants bearing cointegrates of the plasmid in the chromosome were cultured in LB medium. Strains lacking the corresponding genes were selected on LB agar containing 5% (w/v) sucrose and Km, and then verified by PCR, Southern blot hybridization and DNA sequencing.

### RNA preparation and mRNA quantification by real-time PCR

*Pseudomonas putida* cells were treated with RNAprotect Bacteria Reagent (Qiagen, Hilden, Germany) and stored at −20°C until use. Total RNA was prepared from the cells by using an RNeasy Mini kit and an RNase-free DNase set (Qiagen). RNA was determined quantitatively by measuring the absorbance of the diluted samples at 260 nm. One-step real-time RT-PCR was performed using a QuantiTect SYBR Green Kit in the LightCycler Quick system (Roche Diagnostics, Basel, Switzerland) following the manufacturer's instructions. The primers used for RT-PCR are listed in Table S5. Data described in this manuscript are means of at least three independent experiments.

### PAGE, mass analyses and western blot analysis

sodiumdodecyl sulphate polyacrylamide gel electrophoresis (SDS-PAGE) was performed on 10% or 12% polyacrylamide gels and matrix-assisted laser desorption/ionization time-of-flight mass analyses were performed as described elsewhere (Hishinuma et al. 2008). The amount of cells (OD_600_) was defined as the product of the volume (mL) and the optical density (600 nm) of the culture used.

For western blot analysis, protein samples prepared from cell lysates of *P*. *putida* were separated by SDS-PAGE and then transferred to PVDF membranes with an iBlot Dry blotting system (Invitrogen, Carlsbad, CA),and membranes are processed in Can Get Signal solutions (Toyobo, Osaka, Japan) in accordance with the manufacturers’ protocols. Immunological detections of *P*. *putida σ*^32^ and DnaJ were performed using a rabbit polyclonal antibody prepared against *S. marcescens σ*^32^ (Abcam, Cambridge, MA), and an Hsp40 antibody prepared against *E. coli* DnaJ (Abcam), respectively, and visualized with an alkaline phosphatase-conjugated goat anti-rabbit IgG. Detection of alkaline phosphatase activity was carried out in accordance with the instructions on the DIG detection kit (Roche Diagnostics).

### Preparation of insoluble fractions

*Pseudomonas putida* strains were aerobically cultured in 125 mL LB in 500 mL baffled-flasks for overnight. Cells equivalent to 200 OD_600_ units were harvested and suspended in 30 mL of the spent medium. Suspensions were warmed in water bathes at appropriate temperatures for durations indicated with moderate agitation (140 strokes min^−1^), and then bacterial cells were harvested by a brief centrifugation (8730*g* for 5 min at 25°C). Following steps were carried out at 4°C. The cells suspended in 3 mL of suspension buffer (20 mmol/L Tris-Cl containing one tablet of Complete Mini EDTA-free (Roche) per 10 mL, pH8.0) were disrupted five-times by passing through a French pressure cell (800 p.s.i.), and undisrupted cells were removed by centrifugation (2180*g* for 10 min). The insoluble materials and the supernatant, which was used as the soluble fraction, were separated by centrifugation (18,000*g* for 1 h). The insoluble materials was suspended in 3 mL of buffer A (50 mmol/L Tris-Cl, 150 mmol/L NaCl, 1% (v/v) Triton X-100, pH 8.0), and incubated for 2 h. The solution was then centrifuged as above, washed twice with buffer B (50 mmol/L Tris-Cl, 150 mmol/L NaCl, pH 8.0), and suspended in 0.2 mL of a suspension buffer (7 mol/L urea, 2 mol/L thiourea, 100 mmol/L dithiothreitol, 4% [w/v] CHAPS, and 0.2% Bio-lyte 3–10 [Bio-Rad, Hercules, CA]) to prepare the insoluble fraction. The protein concentration of diluted samples was quantified by a protein assay kit (Bio-Rad).

## Results

### Construction of null mutants of *P. putida* ClpB and J-domain protein genes

**Figure 3:**
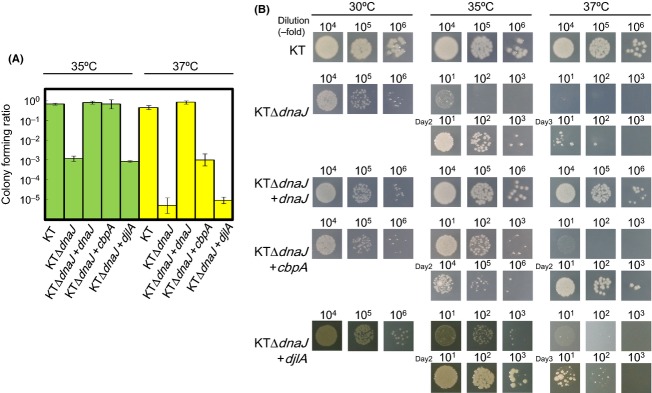
Temperature sensitivity of *Pseudomonas putida* mutant strains. Overnight-grown cells cultured in LB broth were serially diluted 10-fold with LB, and 5 *μ*L of each dilution was then spotted onto prewarmed LB plates-containing streptomycin and incubated at the indicated temperatures. (A) Colony forming ratio at 35°C or 37°C. The wild-type (KT) strain and the *dna*J null-mutant strain (KTΔ*dnaJ*) carried either an empty vector plasmid (pKT231) or a plasmid carrying *dnaJ*, *cbpA*, or *djlA*. Colony numbers formed at 30°C is taken as 1 for each strain. Error bar indicates SD. (B) Photographs of the colonies. Most of the *P. putida* KT2442 *dnaJ* mutant cells could not form colonies on the plate at 35°C or 37°C, and visible colony formation required a few days. The plasmid-borne *dnaJ* efficiently restored the colony-forming ability of the mutant cells at both temperatures. Most of the *P. putida dnaJ* mutant cells carrying a plasmid-borne *cbpA* gene were able to form colonies at 35°C but grew slower than those carrying plasmid-borne *dnaJ*. Essentially the same result was obtained from three independent experiments. Representative photographs, taken after 1 day (not labeled), 2 days (Day2), or 3 days (Day3), are shown.

The *E. coli* DnaK system functions in the disaggregation of thermo-mediated protein aggregates, in cooperation with ClpB. We attempted to construct null mutants of *clpB* and the J-domain protein genes from *P. putida* strain KT and its *dnaK* point mutant strain KT-R2 (R2) to assess their physiological roles. The acquisition of strains that each lacks *clpB* and one of the J-domain protein genes (*dnaJ*, *cbpA*, and *djlA*) would suggest that these genes are dispensable for *P. putida* under certain conditions (Table S1). A *clpB* mutant isolated from KT showed similar growth rates with the parental strain up to 37°C; however, that from R2 showed considerably slower growth at 33°C. Notably, *dnaJ* mutants from both KT and R2 showed retardation of growth at all the temperatures tested. Moreover, KTΔ*dnaJ* was temperature sensitive. Large portions of the population failed to form colonies at 35°C and much less formed at 37°C (Fig. 3). The other J-domain protein gene mutants (KTΔ*cbpA* and KTΔ*djlA*) were not temperature sensitive. The loss of *dnaJ* did not further affect the upper limit of growth temperature in strain R2 (Table S1). On the other hand, deletion of *clpB* and the J-domain protein genes caused marginal effects on their oxidative chemical tolerance (Table S2).

**Figure 4:**
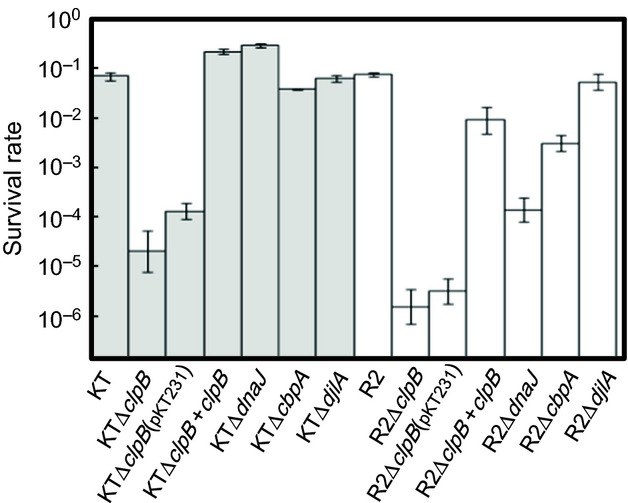
Sensitivity of *Pseudomonas putida* mutants to heat stress. Overnight-grown cells equivalent to an OD_600_ unit of 0.6 were mixed with LB broth in a total volume of 2 mL and incubated at 30°C for five doubling times of the tested strain, to reach the stationary phase. Then, 0.5 mL of the culture was transferred to a 16-mm*φ* glass tube and incubated for 5 min at 50°C. Viable counts were measured after plating serial dilutions of the culture onto LB solid medium at 30°C. The data are the means of three independent experiments. Error bar indicates SD.

We next examined various *P. putida* strains for their sensitivity to high temperatures. For the strains of KT background, about one-tenth of stationary phase cells of the wild-type (KT) and all mutant strains obtained (except for KTΔ*clpB*) retained their colony-forming ability after exposure to 50°C for 5 min (Fig. 4). Deletion of *dnaJ*, which caused growth defect, did not affect the survival rate. For the strains of R2 background, R2, and the R2Δ*djlA* mutant showed survival rates that were similar to that of KT, but the loss of *dnaJ* or *cbpA* reduced the thermotolerance in the strain. Notably, the survival rates of *clpB* mutants isolated from both the KT and R2 strains were much lower than that of the others (Fig. 4). Introduction of plasmid-borne *clpB* recovered their survival rate, indicating that the exogenous *clpB* complemented the gene loss on the chromosome. Preliminary experiments revealed that logarithmically growing cells were considerably more sensitive to the thermal stress than were stationary phase cells, as was observed in *Pseudomonas aeruginosa* (Jørgensen et al. 1999). The thermal sensitivity shown by logarithmically growing cells examined at 45°C again indicated that the *clpB* mutants were quite sensitive to thermal stress (data not shown).

### HSR in *P. putida*

**Figure 5:**
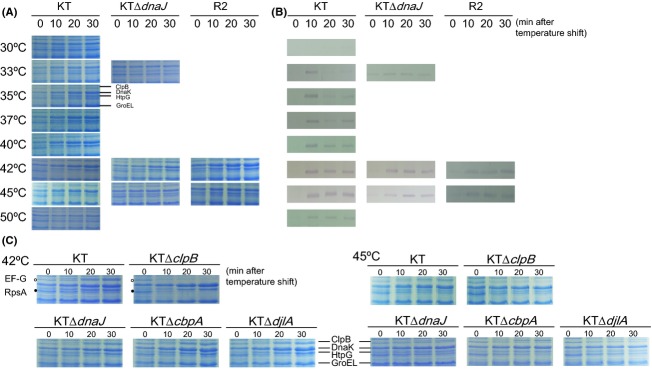
Temperature dependence of the heat shock response. Cells of *Pseudomonas putida* strains were grown in LB broth to the logarithmic growth phase (OD_600_ ˜ 1.0) at 30°C. After a sample was taken at time 0, the culture temperature was shifted to 42°C and samples were taken at every 10 min three times (10, 20, and 30 min). The solubilized proteins were analyzed on 10% SDS-polyacrylamide gels and used for protein staining (prepared from cells in a culture of 100 *μ*L, A) and immunoblotting (prepared from cells in a culture of 50 *μ*L, B). (A) Heat shock response (HSR) in terms of protein levels. Coomassie Brilliant Blue staining of gels showed that the temperature shift induced representative heat shock proteins (ClpB, DnaK, HtpG, and GroEL), which were identified by mass spectrometry. The positions of relevant proteins are marked in the right margin. (B) HSR in terms of *σ*^32^ levels. The cellular level of *σ*^32^ during the temperature up-shift was examined by western blotting with an antiserum against *Serratia marcescens σ*^32^. (C) Effect of gene disruptions on HSR in terms of protein levels. The representative heat shock proteins (Hsps) were induced at 42°C, but not significantly at 45°C. Temperature up-shifts downregulated levels of elongation-factor (EF) G and ribosomal protein S1. The positions of Hsps are shown in the middle margin, and those of EF-G (EF-G, ○) and ribosomal protein S1 (RpsA, •) are shown in the left margin.

We examined whether insertional inactivation mutations of *clpB* and the J-domain protein genes could cause any noticeable changes on the pattern of total cell proteins. Deletion of *clpB*, *cbpA*, and *djlA* apparently did not alter the pattern of cellular proteins in overnight-grown *P. putida* cells, and that of *dnaJ* increased the amounts of DnaK and GroEL slightly in the mutant, but their levels were much less than those in R2 (data not shown). We next monitored the pattern of total cell proteins in the wild-type strain upon various degrees of up-shift of the ambient temperature to examine the effect of the inactivation mutations on the HSR (Fig. 5A). When logarithmically growing cell cultures were transferred from 30°C to 33°C, the increase of Hsps was not significant. At 35°C, slight increases of DnaK, GroEL, and HtpG were detectable, and protein bands for ClpB emerged. Larger up-shifts of temperature induced these proteins further, up to 42°C; however, at 45°C, the amounts of DnaK, GroEL, and HtpG increased for the first 10 min only, whereas that of ClpB seemed to increase continually (Fig. 5A). The increase of Hsps was not obvious at 50°C. The HSR in terms of protein synthesis in *P. putida* mutant strains KTΔ*clpB,* KTΔ*dnaJ,* KTΔ*cbpA,* and KTΔ*djlA* were also examined at 42°C and 45°C (Fig. 5C). All strains exhibited essentially the same response pattern as the wild-type strain at each temperature. We noticed that two major protein species of 84 and 66 kDa were significantly decreased at both temperatures, especially in KTΔ*clpB*. Time-of-flight mass spectrometry analyses revealed that they were elongation factor-(EF)-G and ribosomal protein S1, respectively.

The HSR of *E. coli* is known to be regulated by changes in the concentration of *σ*^32^ (Straus et al. 1987). The temperature-dependent expression of *σ*^32^ was quantified by immunoblotting, where levels in logarithmically growing KT cells were shown to be very low at 30°C and significantly induced within 10 min after transfer to higher temperatures, even at 33°C (Fig. 5B). The *σ*^32^ level decreased thereafter, which is consistent with the presence of a shut-off mechanism. The higher sensitivity of the immunodetection system currently employed caused the shut-off stage at 42°C to be ambiguous. The level of *σ*^32^ was also increased by treatment at 50°C but was probably synthesized during the temperature increase. On the other hand, certain amounts of *σ*^32^ were present in the *dnaJ* mutant and in R2 cells without heat shock. The increase of *σ*^32^ upon temperature shift in the *dnaJ* mutant was not as evident as in the wild-type strain (Fig. 5B).

**Figure 6:**
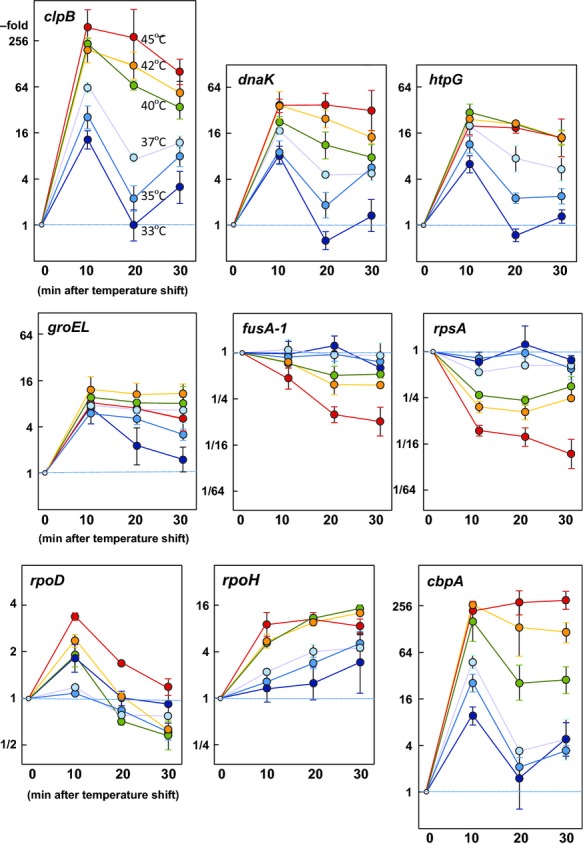
Time course of mRNAs of relevant genes upon heat shock. Cells of *Pseudomonas putida* strains were grown in LB broth to the late logarithmic phase (OD_600_ ˜ 1.0) at 30°C. After a sample was taken at time 0, the culture was shifted to various temperatures, and samples were taken at every 10 min three times (10, 20, and 30 min). Total RNAs were prepared as described in Experimental Procedures. One-*μ*g of total RNA was used to quantify the mRNA level of relevant genes. The relative amount of mRNA (in-fold) was calculated by assuming that one cycle of polymerase chain reaction doubles the amount, and that time 0 is taken as 1 for each gene. Data from at least three replicates are presented. Error bar indicates SD and some of error bars are covered by plot symbols. The raw data are listed in Table S3. The samples were the same as those used for the SDS-PAGE analysis shown in Figure 5. Symbols: dark blue (33°C), blue (35°C), pale blue (37°C), green (40°C), yellow (42°C) and red (45°C).

The temperature-dependent transcriptional responses of four *hsp* genes (*clpB*, *dnaK*, *htpG*, and *groEL*) and two *σ*-factor genes (*rpoD*, which encodes *σ*^70^, and *rpoH*, which encodes *σ*^32^) were examined in parallel by quantifying their mRNA levels in the heat-treated KT cells by qRT-PCR (Fig. 6, Table S3). Increased expressions of the *hsp* genes occurred within 10 min and seemed to be correlated with the level of *σ*^32^ in the cell. Treatments at 40°C, 42°C, and 45°C resulted in the same induction pattern for the *hsp* genes, and their mRNA levels remained high after 30 min. At 33°C, *clpB*, *dnaK*, and *htpG* mRNAs were induced for the first 10 min and decreased rapidly in the next 10 min but then increased again after 30 min; however, this fluctuation was not observed for *groEL*. Higher temperature shifts caused less fluctuation. It should be noted that the fluctuation, which was also seen in the *clpB* mutant (data not shown), was compromised with a depletion of DnaJ (Table S3). The *rpoD* gene was induced during the first 10 min, but thereafter returned to the steady-state level. In contrast, *rpoH* mRNA continued to increase at every temperature, even though the level of *σ*^32^ decreased after transient induction at lower temperatures (Fig. 5B). Levels of the relevant mRNAs in KTΔ*clpB*, KTΔ*dnaJ*, KTΔ*algU*, and R2 cells were also measured at 42°C and 45°C (Fig. S 1, Table S3). Since *P. putida* AlgU belongs to the *σ*^24^ family and is known to be involved in *rpoH* expression (Aramaki et al. 2001), the HSR of KTΔ*algU* was examined. The induction ratios of the *hsp* genes in R2 seemed to be lower due to its high basal expression, but they were generally induced to levels equivalent to or higher than the other strains (Table S3).The expression profiles of the relevant genes were essentially the same in the tested strains at high temperatures, besides that of *rpoH* in KTΔ*algU*. The significant decrease of the *rpoH* mRNA level after prolonged treatment at 45°C indicated that *rpoH* was primary controlled by AlgU at this temperature (Fig. S1, Table S3).

As described above, thermal treatment could downregulate two essential proteins; namely, EF-G and ribosomal proteins S1 (RpsA) (Fig. 5C). The regulation may be controlled at the transcriptional level (Fig. 6, Table S3). EF-G, which is encoded by the *fusA-1* gene (PP0451), belongs to an operon containing the ribosomal protein genes *rpsL* (PP0449) and *rpsG* (PP0450), and the EF-Tu gene *tuf-2* (PP0452). A comprehensive microarray analysis has confirmed the operon structure of the ribosomal protein genes, and about two-thirds of them, including *rpsA* (PP1772), were highly expressed in *P. putida* (Frank et al. 2011). We also quantified mRNAs for *rplB* (PP0457, encoding ribosomal protein L2) and *rpsE* (PP0471, encoding ribosomal protein S5) at 42°C and 45°C, to assess if an acute downregulation at higher temperatures is specific for *rpsA* (Table S3). The temperature up-shift to 42°C downregulated *rpsE* slightly, and so did *rplB* as for *fusA-1*, albeit to lesser extents than for *rpsA*. These genes were significantly downregulated at 45°C, as in the case of *rpsA*.

### *Pseudomonas putida* cbpA is heat inducible

Canonical *E. coli hsp* genes (or the uppermost gene when one forms an operon) possess typical heat shock promoter sequences, which can be recognized by the heat shock *σ*-factor *σ*^32^ (Koo et al. 2009). A comprehensive study revealed that more than 50 transcriptional units are under the control of *σ*^32^, and about two-thirds of the *σ*^32^ promoters are localized within 100 bases upstream of the initiation codon of their uppermost gene in *E. coli* (Nonaka et al. 2006). We have searched for possible *σ*^32^-dependent genes (of which the promoter region is situated in close proximity) that have a strong sequence similarity to the proposed consensus sequence for *σ*^32^ promoters (CTTGAA−N13-17−CCCCATNT; Yura et al. 2000) in the *P*. *putida* genome sequence (Nelson et al. 2002). Consequently, in addition to the previously identified *σ*^32^-dependent genes, *cbpA*, *hfq* (PP4894 encoding the host factor-I), and *secA* (PP1345 encoding the SecA subunit of preprotein translocase) were shown to possess potential *σ*^32^ promoter sequences (Fig. S2). Coincidentally, *secA* and *cbpA* have identical sequences for their −35 and −10 regions, as well as the spaces between these regions. *E. coli* CbpA is a J-domain protein with an ability to stimulate the ATPase activity of DnaK (Hennessy et al. 2005), but it is not an Hsp (Yamashino et al. 1994). The expression of its encoding gene is mediated by *σ*^S^, an alternative *σ*-factor, in the early stationary phase or under phosphate starvation. We quantitated the mRNA levels of *cbpA* in relation to its response to heat shock in *P. putida*. In the logarithmic growth phase, the basic expression of *cbpA* was considerably lower than that of the other *hsp* genes, and even lower than that of *clpB* (Table S3), but its expression was induced by two orders of magnitude upon subjection to high temperatures (Fig. 6). The *cbpA* gene was similarly induced in *rpoS* mutant cells upon heat shock, but was not increased in stationary phase KT cells at normal temperature (data not shown).

### Disaggregation of heat-mediated protein aggregates

**Figure 7:**
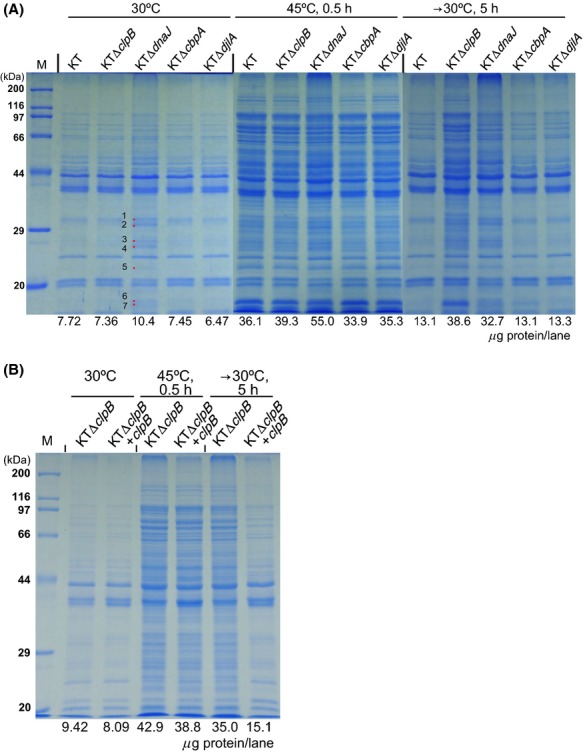
Role of ClpB in the solubilization of thermo-mediated protein aggregates in *Pseudomonas putida*. Cells of *P. putida* strains were grown overnight at 30°C, and two aliquots were further cultured at 45°C for 30 min. One of the two aliquots that had been cultured at 45°C was further cultured at 30°C for 5 h. Insoluble proteins were prepared as described in Experimental Procedures. Fractions corresponding to identical cell masses (based on the optical density) were analyzed by SDS-PAGE (12% gel), and the proteins were visualized with Coomassie Brilliant Blue. The amount of protein loaded is shown below each lane. (A) ClpB was essential for the solibilization of thermo-mediated protein aggregates in *P. putida*. Proteins identified by time-of-flight mass spectrometry in the *dnaJ* mutant cells: 1, 50S ribosomal protein L2; 2, 30S ribosomal protein S2; 3, 30S ribosomal protein S3; 4, mixture of 50S ribosomal protein L3 and 30S ribosomal protein S4; 5, 50S ribosomal protein L5; 6, 50S ribosomal protein L16; and 7, 30S ribosomal protein S9. (B) The introduction of plasmid-borne *clpB* into the *clpB* mutant fully restored the disaggregation ability of the host cells. The *clpB* null-mutant strain (KTΔ*clpB*) carried an empty vector plasmid (pKT231).

The formation and disaggregation of heat-mediated protein aggregates in *P. putida* strains were examined in our study. Overnight-grown KT cells were exposed to various temperatures (from 30°C to 45°C) for 30 min to determine if thermal treatment could cause the formation of protein aggregates (Fig. S3). Compared with proteins in the insoluble fraction of KT cells grown at 30°C, which remained nonaggregated, some small-sized protein aggregates, (named hereafter as aggregated proteins) formed in the heat-stressed cultures, even at 37°C. The number and amount of aggregated proteins increased gradually upon temperature increase. A temperature shift-back to 30°C for less than 2 h allowed significant decreases of the thermo-mediated protein aggregates. The formation and decrease of protein aggregates in *P. putida* KTΔ*clpB* and in the three J-domain protein gene mutants were also examined (Fig. 7). At 30°C, the KTΔ*dnaJ* strain alone accumulated several unique insoluble proteins, which were subsequently found to be ribosomal proteins (Fig. 7A). Exposure to 45°C for 30 min resulted in protein aggregate formation in every strain. The amounts of protein aggregates formed in the wild-type, KTΔ*clpB*, KTΔ*cbpA*, and KTΔ*djlA* cells were quite similar; however, clearly more aggregates were formed in the *dnaJ* mutant, especially those with a high-molecular-mass (>200 kDa). Although a certain amount of protein aggregates that had formed in the *dnaJ* mutant cells had disappeared, those in the *clpB* mutant cells were virtually not solubilized during the recovery phase. A deficiency in *cbpA* or *djlA* had no effect on the formation and removal of protein aggregates. The introduction of pKT231-borne *clpB* into the *clpB* mutant cells (Fig. 7B), and that of pKT231-borne *dnaJ* into the *dnaJ* mutant cells (Fig. S4), allowed recovery of the protein aggregate clearance ability, indicating that these genes complemented the corresponding gene defects in the chromosome. Meanwhile, DnaK should also be essential for the solubilization of thermo-mediated protein aggregates, as aggregates that had formed in the *dnaK* point mutant R2 cells were not solubilized (Fig. S5). Essentially, the same result was obtained for R2Δ*clpB* cells, but a slight decrease of aggregated proteins was observed in the *dnaJ* mutant cells.

### *Pseudomonas putida* CbpA can partially substitute the functions of DnaJ

The DnaJ analogs CbpA and DjlA are known to support cell growth (Ueguchi et al. 1995; Genevaux et al. 2001) and the disaggregation of thermo-mediated protein aggregates (Gur et al. 2004) in *E. coli dnaJ* mutants. *P. putida* DnaJ and CbpA share an overall amino acid identity of 38%, and their J-domains, which contain a highly conserved His-Pro-Asp tripeptide thought to be involved in substrate binding, are about 60% identical. Meanwhile, *P. putida* CbpA possesses an obvious Gly/Phe-rich domain, similar to that of DnaJ (Fig. 2). In contrast, *P. putida* DjlA shows partial similarity with DnaJ or CbpA only in its J-domain, and is merely 30% identical to *E. coli* DjlA. To assess whether *P. putida* CbpA and/or DjlA functions as well as DnaJ in cell growth at high temperatures, plasmid-borne *dnaJ*, *cbpA*, or *djlA* was independently introduced into the *dnaJ* mutant cells (Fig. 3). The introduction of *djlA* only slightly supported the cell growth at 35°C, and did not show any effect at 37°C. Although a longer incubation time was required, the exogenous *cbpA* allowed every KTΔ*dnaJ* cell colony to form at 35°C, suggesting that CbpA can partially substitute DnaJ in functions relating to colony formation. At 37°C, only small fractions of the *dnaJ* mutant cells were able to form colonies in the presence of a *cbpA* plasmid, indicating that *dnaJ* has an exclusive function(s) for the cell growth of *P. putida* at high temperatures. On the other hand, CbpA may play an equivalent role to DnaJ in the prevention of heat-mediated protein aggregates since the introduction of *cbpA* on a plasmid mitigated aggregate formation (Fig. S4). About one-half of the thermo-mediated protein aggregates were solubilized in KTΔ*dnaJ* during the recovery stage. Chromosomally encoded CbpA would be present in the *dnaJ* mutant and production of extra CbpA from the plasmid might improve solubilization of aggregates.

## Discussion

We have described herein the transcriptional and translational HSR of *P. putida*. It is likely that the strain has an *E. coli*-type system that controls the activity and quantity of *σ*^32^, which thereby controls the HSR, but the two bacterial systems are not identical. As described previously, *P. putida* DnaK plays a major role in the control of *σ*^32^ (Kobayashi et al. 2011). DnaK, DnaJ, and GrpE comprise the so-called DnaK system, which is a key component of the chaperone networks that are involved in facilitating cellular proteostasis (Gamer et al. 1996; Calloni et al. 2012). A depletion of either the DnaK system or GroEL/S is known to cause an accumulation of Hsps in *E. coli* (Tomoyasu et al. 1998; Guisbert et al. 2008), as well as in the *dnaK* mutant *P. putida* strain R2 in the stationary phase (Kobayashi et al. 2011). However, the *P. putida dnaJ* null mutant did not accumulate Hsps as much as did R2 under normal growth conditions. We hypothesize that CbpA acts as a de facto backup J-domain protein for the control of the HSR. *P. putida* CbpA has a considerably high estimated isoelectric point (pI ˜ 9.6), yet it is 67% identical to the *E. coli* CbpA. *P. putida* CbpA has remarkable sequence similarities with DnaJ from the same strain, especially in helices II and III of the J-domain (Fig. 2). The absence of DnaJ should transiently permit an increase of *σ*^32^, which causes the induction of *hsp* genes. If, as in *E. coli*, *P. putida cbpA* were controlled by *σ*^s^, the negative feedback loop-mediated shut-off of the HSR would not be switched on; however, *P. putida cbpA* is most likely controlled by *σ*^32^. CbpA, which may share substrate proteins with DnaJ, likely coordinates the binding of *σ*^32^ for DnaK, sequestering *σ*^32^ from RNA polymerase, and therefore the expression of *hsp* genes would be repressed. Indeed, the mRNA levels of the *dnaJ* mutant were not significantly different from the other cultures (Table S3). Since a certain amount of *σ*^32^ and more DnaK and GroEL were found in the *dnaJ* mutant cells, CbpA might be less functional than DnaJ in the FtsH-mediated degradation of *σ*^32^, or DnaJ may be unique for the process.

The high thermal sensitivities of the *clpB* mutants may indicate that the cell survival after harsh heat stress is dependent on the protein disaggregation ability of the cell. It has been suggested that the removal of protein aggregates by degradation is not sufficient to ensure cell survival during severe heat stress (Weibezahn et al. 2004).The DnaK system and ClpB cooperatively solubilize previously aggregated proteins (Glover and Lindquist 1998; Goloubinoff et al. 1999). Protein aggregates formed in the *dnaK* point mutant R2 cells and R2Δ*clpB* mutant cells were not solubilized at all (Fig. S5); however, inconsistent with the inability of solubilization, stationary phase R2 cells were much more tolerant than the *clpB* mutant cells with regard to their survival rate (Fig. 4). This observation indicates that ClpB may be involved in a protective mechanism against thermal stress other than the solubilization of protein aggregates. On the other hand, DnaJ is considered to be the primary J-domain protein for the solubilization of thermo-mediated protein aggregates. Although considerably more aggregates were formed in the *dnaJ* mutants, they were far more tolerant to thermal stress (Fig. 4). ClpB is probably essential for the renaturation of proteins, but the function of DnaJ in the solubilization of the protein aggregate may be replaced by CbpA. We noticed that high-molecular-weight aggregates were formed in the *dnaJ* mutant cells and were apparently not solubilized. *E. coli* DnaJ has been shown to mediate DnaK binding to large protein aggregates (Acebrón et al. 2008). The protein aggregates formed in cells of R2 background were considerably fewer than that in the wild-type cells. The high level of Hsps (i.e., GroEL/S and DnaJ) probably protects proteins from aggregation, as observed in *E. coli* (Gragerov et al. 1992). We do not know whether HtpG and the mutated DnaK contribute to the protection in *P. putida*.

Numerous cellular proteins, including many essential proteins such as subunits of RNA polymerase and EF-G, have been identified to be prone to aggregation in heat-treated *E. coli* cells (Mogk et al. 1999). We have identified a number of proteins in the insoluble fraction of heat-treated KTΔ*clpB* mutant cells. In addition to EF-G, some ribosomal proteins were prone to be aggregated (Fig. S3). Several ribosomal proteins were also found to be aggregated in overnight-grown KTΔ*dnaJ* cells (Fig. 7). In *P. putida*, exposure to high temperatures downregulated *rpsA* (Fig. 6, Table S3), encoding ribosomal protein S1, which is required for the translation of most mRNAs in *E. coli* (Sørensen et al. 1998), along with some ribosomal protein genes (Table S3), as observed for *E*. *coli* (Wade et al. 2006). Since the unrestricted synthesis of thermolabile proteins can potentially lead the cell to danger under harsh conditions, *P. putida* might arrest the de novo protein synthesis of non-Hsps (and probably Hsps as well) upon exposure to high temperatures. In addition, *hsp* gene mRNAs, which were abundant in cells at 45°C, may saturate pre-existing ribosomes and hinder the synthesis of non-Hsps.

The temperature at which cells reach the maximum level of Hsp synthesis may depend on the organisms and as well as pre-shift temperatures, where a 10°C to 12°C increase from optimal growth temperature is generally required for full induction (Yamamori and Yura 1980; Key et al. 1981). As described above, a mere 3°C up-shift of ambient temperature (from 30°C to 33°C), would be unlikely to give rise to a high accumulation of incorrectly folded protein, and yet it still caused a sharp induction of the *hsp* genes (Fig. 6). We have wondered how *P. putida* cells could sense such a subtle change of temperature. Many previously obtained experimental data support the conclusion that in *E. coli,* the translation control of *rpoH* mRNA responds more directly to changes in temperature than to sensing the cellular folding environment that results from the temperature change (Guisbert et al. 2008; Kortmann and Narberhaus 2012). However, the translational upregulation may not fully explain the acute increase of active *σ*^32^. How can *σ*^32^ be released from the DnaK system? An intriguing report that describes the localization of *σ*^32^ on the inner membrane has been recently published (Lim et al. 2013), where *σ*^32^ was shown to be directed by the signal recognition particle (SRP) and SRP receptor to the membrane surface, and then was subjected to chaperone-mediated activity control and FtsH-mediated degradation control. The revised model explains well how *σ*^32^ homeostatic control senses a subtle change of ambient temperature, which may not cause the accumulation of unfolded or misfolded proteins but may result in membrane perturbation. The activity control on the border would be an efficient way to perceive the state of cytosolic and inner membrane proteostasis.

We have described the HSR and chaperone-mediated solubilization of protein aggregates in *P. putida*. The strain employs principally the same system as that in *E. coli* to control the activity and quantity of *σ*^32^ and the solubilization of protein aggregates. However, the regulation of the J-domain protein CbpA was distinct in the two strains, suggesting a unique role for the protein. Further investigations of the regulation of stress response in *P. putida* will provide us with new insights into the divergence in bacterial physiology.

## Supporting Information

**Figure S1.** Time course of heat shock response in the *hsp* gene expression. Cells of *Pseudomonas putida* strains were grown and treated as described in the legend for Figure 6. Changes in the mRNA level are shown by yellow (42°C) and red (45°C) symbols. The temperature up-shift similarly upregulated the mRNA levels of representative *hsp* genes (*clpB, dnaK*, *htpG*, and *groEL*) in every strain. The level of *clpB* was able to be quantified in the *clpB* mutant as a 5′ portion of the gene was remained to be intact. A harsh heat stress significantly downregulated the mRNA levels of the EF-G gene (*fusA-1*) and a ribosomal protein gene (*rpsA*). The relative amount of mRNA (in -fold) was calculated by assuming that one cycle of polymerase chain reaction doubles the amount, and that time 0 is taken as 1 for each gene. Data from at least three replicates are presented. Error bars are not shown to simplify the figure. **Figure S2.** Alignment of the 5′-untranslated region of *σ*^32^-dependent genes of *Pseudomonas putida*. Promoter sequences (−35 and −10 regions) are highlighted in red. **Figure S3.** Temperature-dependent protein aggregation in *Pseudomonas putida* cells. *P. putida* KT2442 wild-type cells were grown overnight at 30°C and then further cultured at the indicated temperatures for 30 min. Two aliquots that had been culture at 45°C were further cultured at 30°C for 2 or 5 h. Overnight-grown *P. putida* KT2442 Δ*clpB* cells were cultured at 45°C for 30 min and then at 30°C for 5 h. Insoluble proteins were prepared as described in Experimental procedures. Fractions corresponding to identical cell masses (based on the optical density) were analyzed by SDS-PAGE (12% gel), and the proteins were visualized with Coomassie brilliant blue. Proteins identified by mass spectrometry are listed in the left (membrane proteins) and right (probable aggregated proteins except N) margins. **Figure S4.** Role of *Pseudomonas putida cbpA* in the disaggregation of protein aggregates. *P. putida* cells were grown and treated as described in the legend of Figure S3. The introduction of plasmid-borne *dnaJ* into the *dnaJ* mutant fully restored the disaggregation ability of the host cells, and the introduction of *cbpA* protected the cells from protein aggregate formation and probably supported the disaggregation process. **Figure S5.** Evaluation of the disaggregation in R2 and its *clpB* and *dnaJ* mutants. *Pseudomonas putida* cells were grown and treated as described in the legend of Figure S3. Protein aggregates that had formed in R2 and its *clpB* and *dnaJ* mutants were hardly disaggregated.

**Table S1.** Doubling time of *Pseudomonas putida* KT2442 strains under various temperature. **Table S2.** Chemical growth inhibition of *Pseudomonas putida* KT2442 strains.

Table S3 Time course quantification of relevant genes by real-time RT-PCR.

Table S4 Bacteria and plasmids used in the study.

Table S5 Primers used in this study.
