# Supplementary material for: Genetic and phenotypic characterization of the heat shock response in Pseudomonas putida
Source: Microbiologyopen. 2014 Oct 10;3(6):922–36. doi: 10.1002/mbo3.217 (PMC4263515; doi:10.1002/mbo3.217)
Supplement: Supplementary file 1 — Figure S1. Time course of heat shock response in the hsp gene expression. Cells of Pseudomonas putida strains were grown and treated as described in the legend for Figure 6. Changes in the mRNA level are shown by yellow (42°C) and red (45°C) symbols. The temperature up-shift similarly upregulated the mRNA levels of representative hsp genes (clpB, dnaK, htpG, and groEL) in every strain. The level of clpB was able to be quantified in the clpB mutant as a 5′ portion of the gene was remained to be intact. A harsh heat stress significantly downregulated the mRNA levels of the EF-G gene (fusA-1) and a ribosomal protein gene (rpsA). The relative amount of mRNA (in -fold) was calculated by assuming that one cycle of polymerase chain reaction doubles the amount, and that time 0 is taken as 1 for each gene. Data from at least three replicates are presented. Error bars are not shown to simplify the figure. Figure S2. Alignment of the 5′-untranslated region of σ32-dependent genes of Pseudomonas putida. Promoter sequences (−35 and −10 regions) are highlighted in red. Figure S3. Temperature-dependent protein aggregation in Pseudomonas putida cells. P. putida KT2442 wild-type cells were grown overnight at 30°C and then further cultured at the indicated temperatures for 30 min. Two aliquots that had been culture at 45°C were further cultured at 30°C for 2 or 5 h. Overnight-grown P. putida KT2442 ΔclpB cells were cultured at 45°C for 30 min and then at 30°C for 5 h. Insoluble proteins were prepared as described in Experimental procedures. Fractions corresponding to identical cell masses (based on the optical density) were analyzed by SDS-PAGE (12% gel), and the proteins were visualized with Coomassie brilliant blue. Proteins identified by mass spectrometry are listed in the left (membrane proteins) and right (probable aggregated proteins except N) margins. Figure S4. Role of Pseudomonas putida cbpA in the disaggregation of protein aggregates. P. putida cells were grown and treated a [file mbo30003-0922-sd1.pptx]

## Slide 1
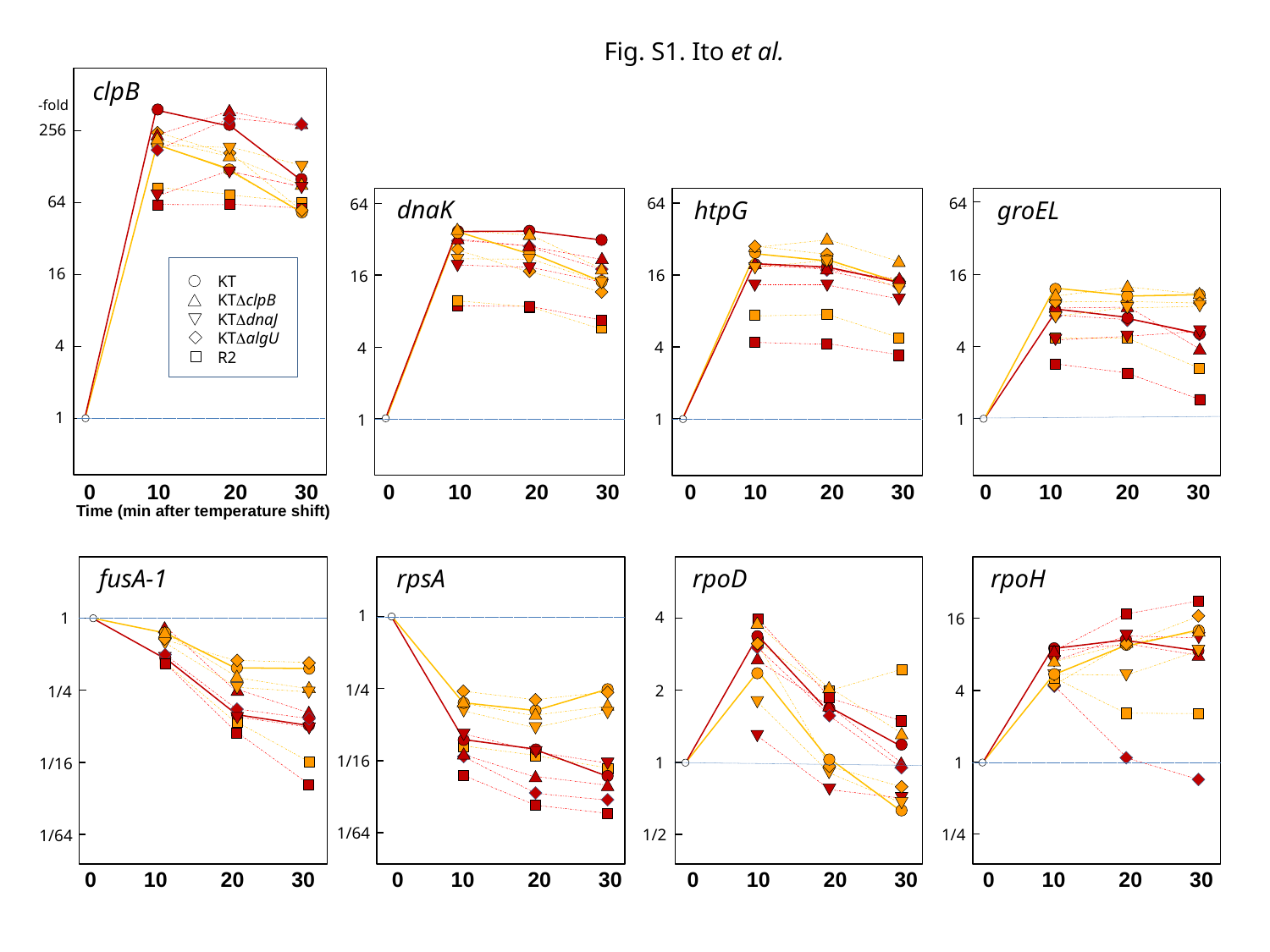

Fig. S1. Ito et al.
clpB
-fold
256
 64
 16
 4
 1
 64
 16
 4
 1
 64
 16
 4
 1
 64
 16
 4
 1
dnaK
groEL
htpG
KT
KTDclpB
KTDdnaJ
KTDalgU
R2
0 10 20 30 0 10 20 30 0 10 20 30 0 10 20 30
Time (min after temperature shift)
 16
 4
 1
 1/4
fusA-1
rpsA
rpoD
rpoH
 1
 1/4
 1/16
 1/64
 4
 2
 1
 1/2
 1
 1/4
 1/16
 1/64
0 10 20 30 0 10 20 30 0 10 20 30 0 10 20 30

## Slide 2
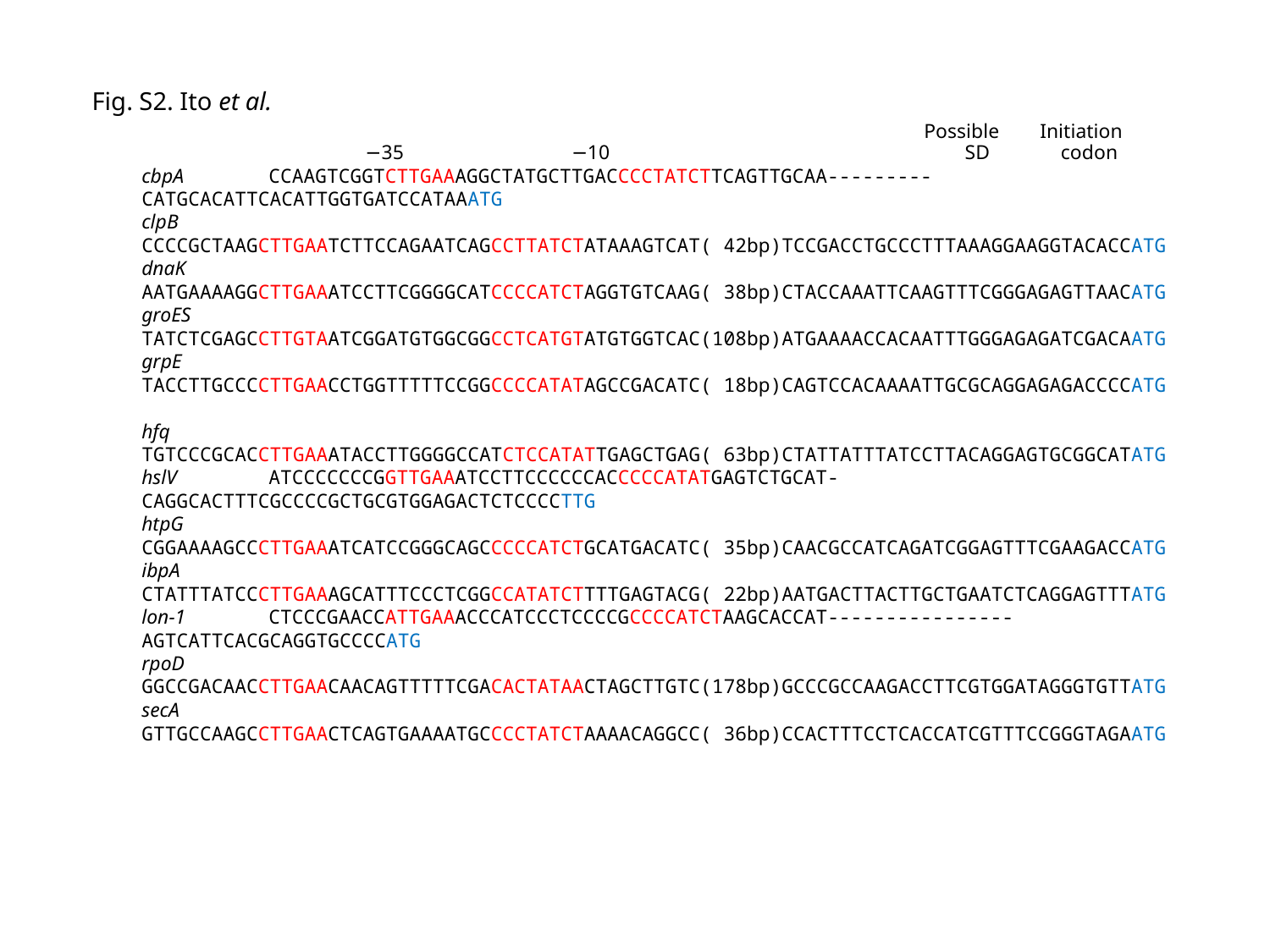

Fig. S2. Ito et al.
 Possible Initiation
 −35 −10 SD codon
cbpA 	CCAAGTCGGTCTTGAAAGGCTATGCTTGACCCCTATCTTCAGTTGCAA---------CATGCACATTCACATTGGTGATCCATAAATG
clpB 	CCCCGCTAAGCTTGAATCTTCCAGAATCAGCCTTATCTATAAAGTCAT( 42bp)TCCGACCTGCCCTTTAAAGGAAGGTACACCATG
dnaK 	AATGAAAAGGCTTGAAATCCTTCGGGGCATCCCCATCTAGGTGTCAAG( 38bp)CTACCAAATTCAAGTTTCGGGAGAGTTAACATG
groES 	TATCTCGAGCCTTGTAATCGGATGTGGCGGCCTCATGTATGTGGTCAC(108bp)ATGAAAACCACAATTTGGGAGAGATCGACAATG
grpE	TACCTTGCCCCTTGAACCTGGTTTTTCCGGCCCCATATAGCCGACATC( 18bp)CAGTCCACAAAATTGCGCAGGAGAGACCCCATG
hfq	TGTCCCGCACCTTGAAATACCTTGGGGCCATCTCCATATTGAGCTGAG( 63bp)CTATTATTTATCCTTACAGGAGTGCGGCATATG
hslV 	ATCCCCCCCGGTTGAAATCCTTCCCCCCACCCCCATATGAGTCTGCAT-CAGGCACTTTCGCCCCGCTGCGTGGAGACTCTCCCCTTG
htpG	CGGAAAAGCCCTTGAAATCATCCGGGCAGCCCCCATCTGCATGACATC( 35bp)CAACGCCATCAGATCGGAGTTTCGAAGACCATG
ibpA	CTATTTATCCCTTGAAAGCATTTCCCTCGGCCATATCTTTTGAGTACG( 22bp)AATGACTTACTTGCTGAATCTCAGGAGTTTATG
lon-1	CTCCCGAACCATTGAAACCCATCCCTCCCCGCCCCATCTAAGCACCAT----------------AGTCATTCACGCAGGTGCCCCATG
rpoD 	GGCCGACAACCTTGAACAACAGTTTTTCGACACTATAACTAGCTTGTC(178bp)GCCCGCCAAGACCTTCGTGGATAGGGTGTTATG
secA 	GTTGCCAAGCCTTGAACTCAGTGAAAATGCCCCTATCTAAAACAGGCC( 36bp)CCACTTTCCTCACCATCGTTTCCGGGTAGAATG

## Slide 3
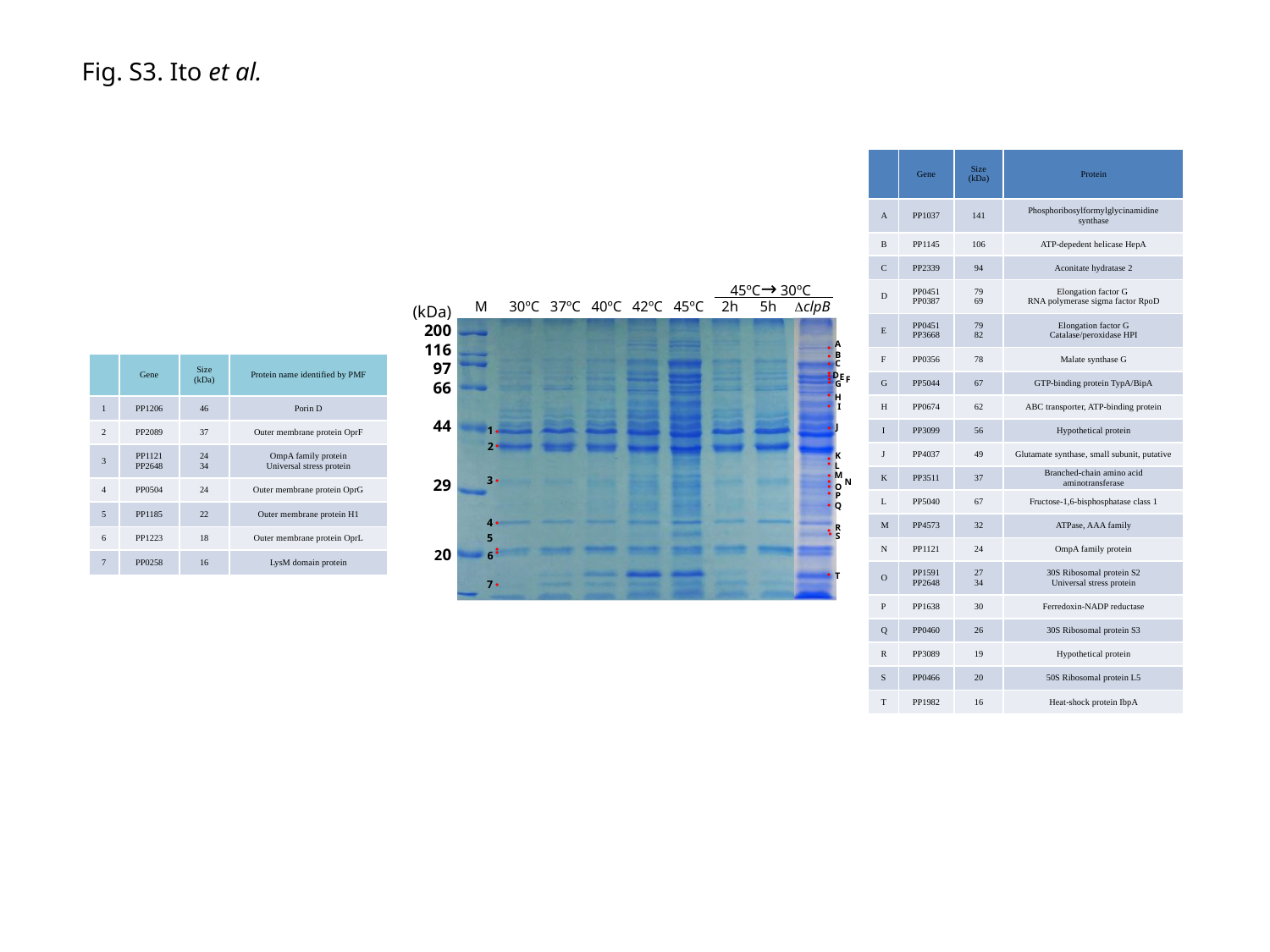

Fig. S3. Ito et al.
| | Gene | Size (kDa) | Protein |
| --- | --- | --- | --- |
| A | PP1037 | 141 | Phosphoribosylformylglycinamidine synthase |
| B | PP1145 | 106 | ATP-depedent helicase HepA |
| C | PP2339 | 94 | Aconitate hydratase 2 |
| D | PP0451 PP0387 | 79 69 | Elongation factor G RNA polymerase sigma factor RpoD |
| E | PP0451 PP3668 | 79 82 | Elongation factor G Catalase/peroxidase HPI |
| F | PP0356 | 78 | Malate synthase G |
| G | PP5044 | 67 | GTP-binding protein TypA/BipA |
| H | PP0674 | 62 | ABC transporter, ATP-binding protein |
| I | PP3099 | 56 | Hypothetical protein |
| J | PP4037 | 49 | Glutamate synthase, small subunit, putative |
| K | PP3511 | 37 | Branched-chain amino acid aminotransferase |
| L | PP5040 | 67 | Fructose-1,6-bisphosphatase class 1 |
| M | PP4573 | 32 | ATPase, AAA family |
| N | PP1121 | 24 | OmpA family protein |
| O | PP1591 PP2648 | 27 34 | 30S Ribosomal protein S2 Universal stress protein |
| P | PP1638 | 30 | Ferredoxin-NADP reductase |
| Q | PP0460 | 26 | 30S Ribosomal protein S3 |
| R | PP3089 | 19 | Hypothetical protein |
| S | PP0466 | 20 | 50S Ribosomal protein L5 |
| T | PP1982 | 16 | Heat-shock protein IbpA |
 45ºC→ 30ºC
M 30ºC 37ºC 40ºC 42ºC 45ºC 2h 5h DclpB
(kDa)
200
116
97
66
44
29
20
A
●
B
●
C
| | Gene | Size (kDa) | Protein name identified by PMF |
| --- | --- | --- | --- |
| 1 | PP1206 | 46 | Porin D |
| 2 | PP2089 | 37 | Outer membrane protein OprF |
| 3 | PP1121 PP2648 | 24 34 | OmpA family protein Universal stress protein |
| 4 | PP0504 | 24 | Outer membrane protein OprG |
| 5 | PP1185 | 22 | Outer membrane protein H1 |
| 6 | PP1223 | 18 | Outer membrane protein OprL |
| 7 | PP0258 | 16 | LysM domain protein |
●
D
●
E
●
F
●
G
●
H
●
I
●
J
1
●
●
2
●
K
●
L
●
M
●
3
N
●
●
O
●
P
●
Q
●
4
●
R
●
S
5
●
●
6
●
T
●
7
●

## Slide 4
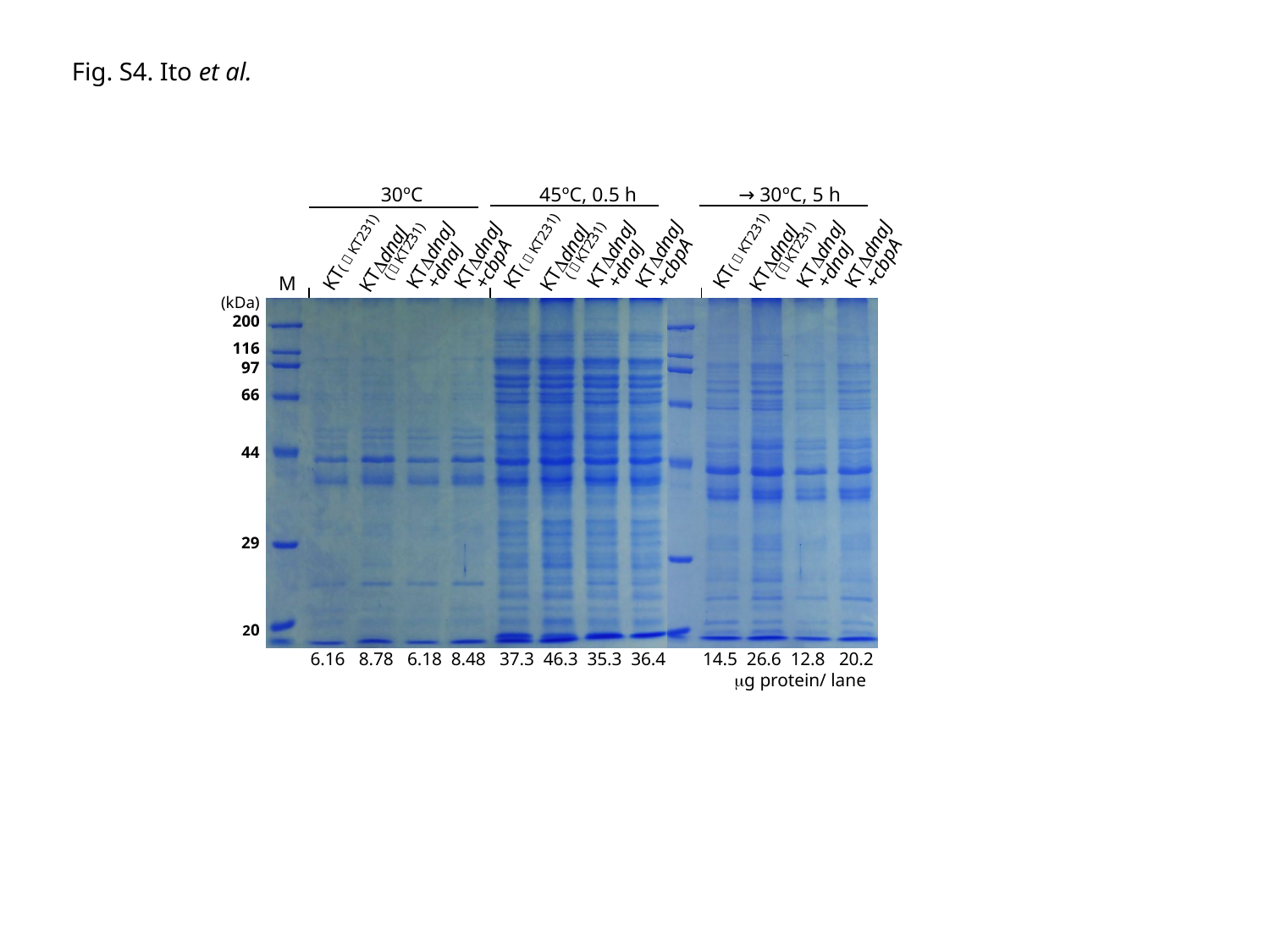

Fig. S4. Ito et al.
30ºC 45ºC, 0.5 h → 30ºC, 5 h
KTDdnaJ
 (ｐKT231)
KTDdnaJ
 (ｐKT231)
KTDdnaJ
 (ｐKT231)
KT(ｐKT231)
KT(ｐKT231)
KT(ｐKT231)
KTDdnaJ
 +cbpA
KTDdnaJ
 +cbpA
KTDdnaJ
 +dnaJ
KTDdnaJ
 +dnaJ
KTDdnaJ
 +cbpA
KTDdnaJ
 +dnaJ
M
(kDa)
200
116
97
66
44
29
20
 6.16 8.78 6.18 8.48 37.3 46.3 35.3 36.4 14.5 26.6 12.8 20.2
 mg protein/ lane

## Slide 5
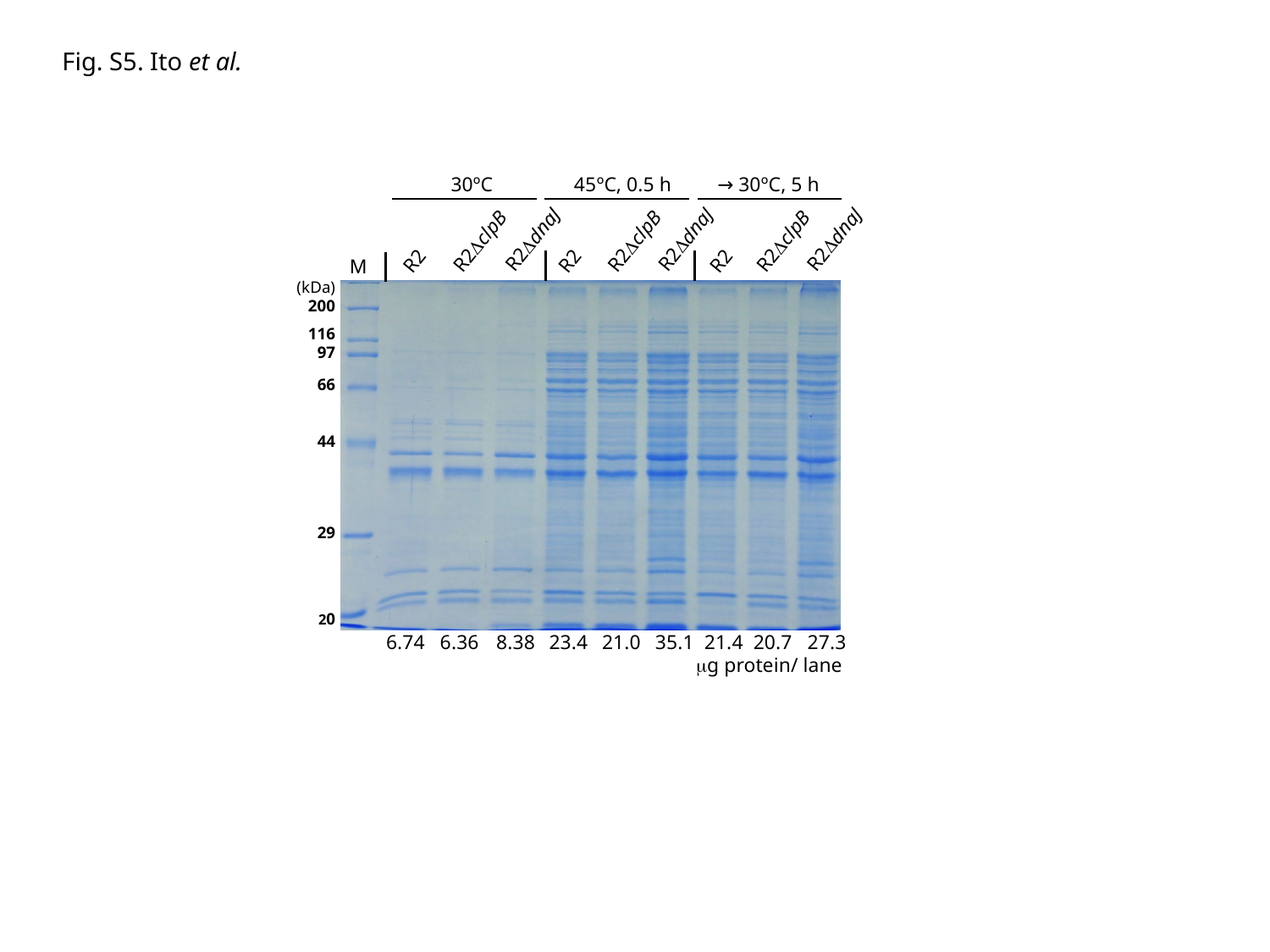

Fig. S5. Ito et al.
30ºC 45ºC, 0.5 h → 30ºC, 5 h
R2DdnaJ
R2DdnaJ
R2DdnaJ
R2DclpB
R2DclpB
R2DclpB
R2
R2
R2
M
(kDa)
200
116
97
66
44
29
20
6.74 6.36 8.38 23.4 21.0 35.1 21.4 20.7 27.3
 mg protein/ lane
