# Supplementary figures and images for: Genetic and phenotypic characterization of the heat shock response in Pseudomonas putida
Source: Microbiologyopen. 2014 Oct 10;3(6):922–36. doi: 10.1002/mbo3.217 (PMC4263515; doi:10.1002/mbo3.217)

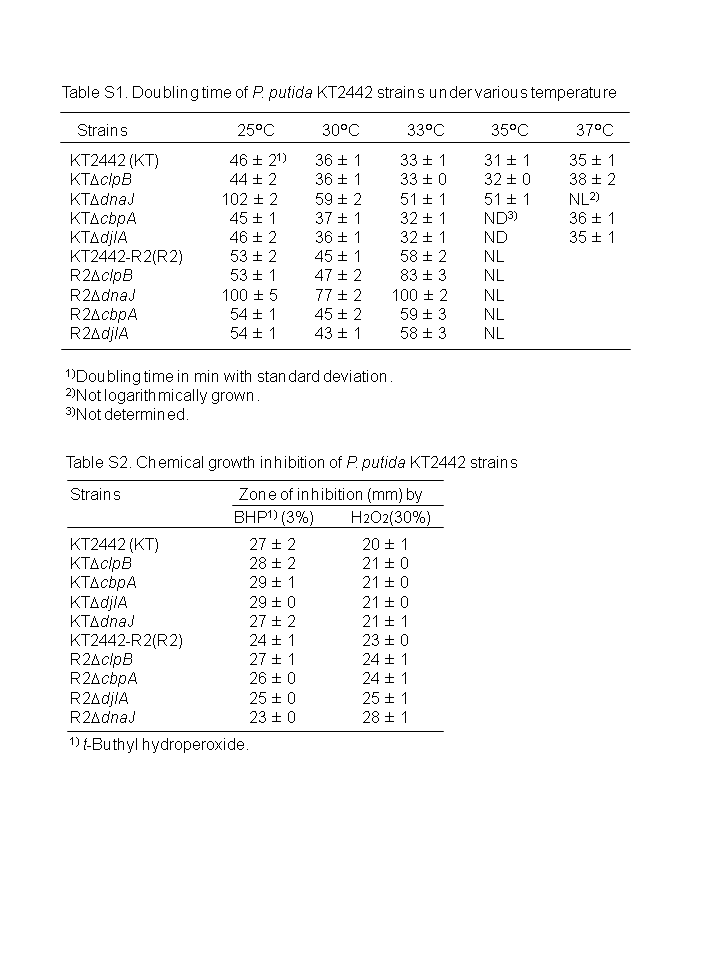

Supplement: Supplementary file 2 — Table S1. Doubling time of Pseudomonas putida KT2442 strains under various temperature. Table S2. Chemical growth inhibition of Pseudomonas putida KT2442 strains. [file mbo30003-0922-sd2.tif]

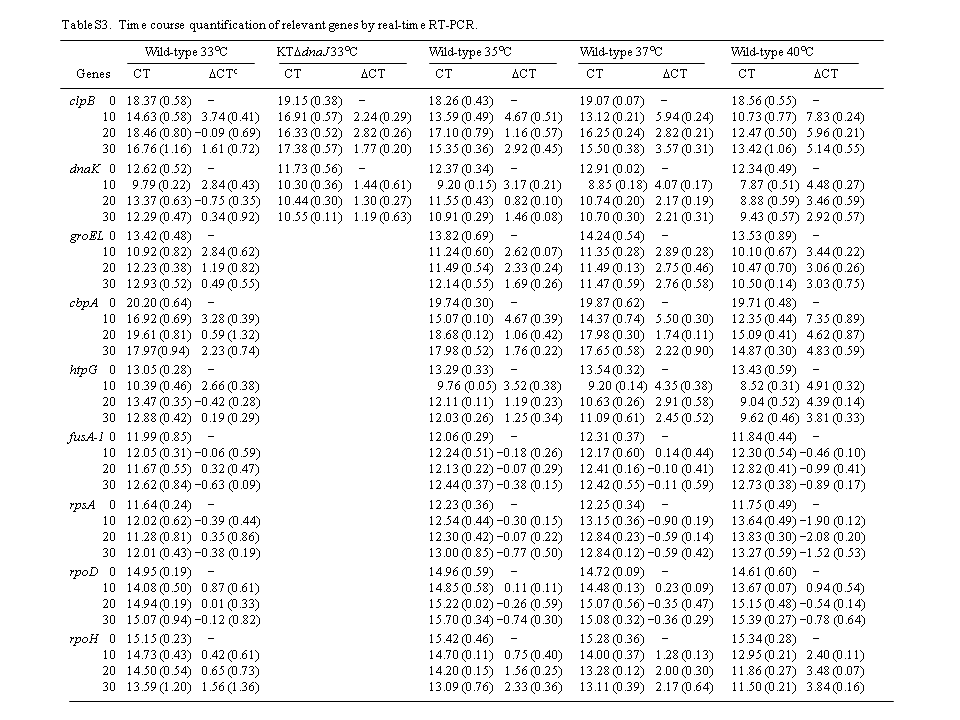

Supplement: Table S3 — Time course quantification of relevant genes by real-time RT-PCR. [file mbo30003-0922-sd3.tif]

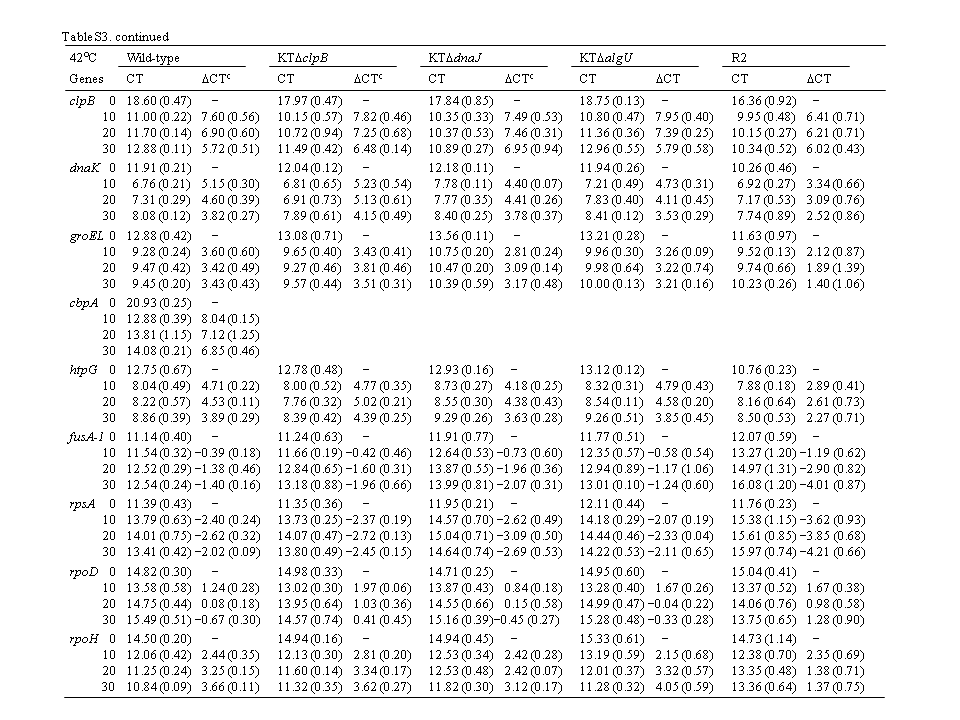

Supplement: Supplementary file 4 [file mbo30003-0922-sd4.tif]

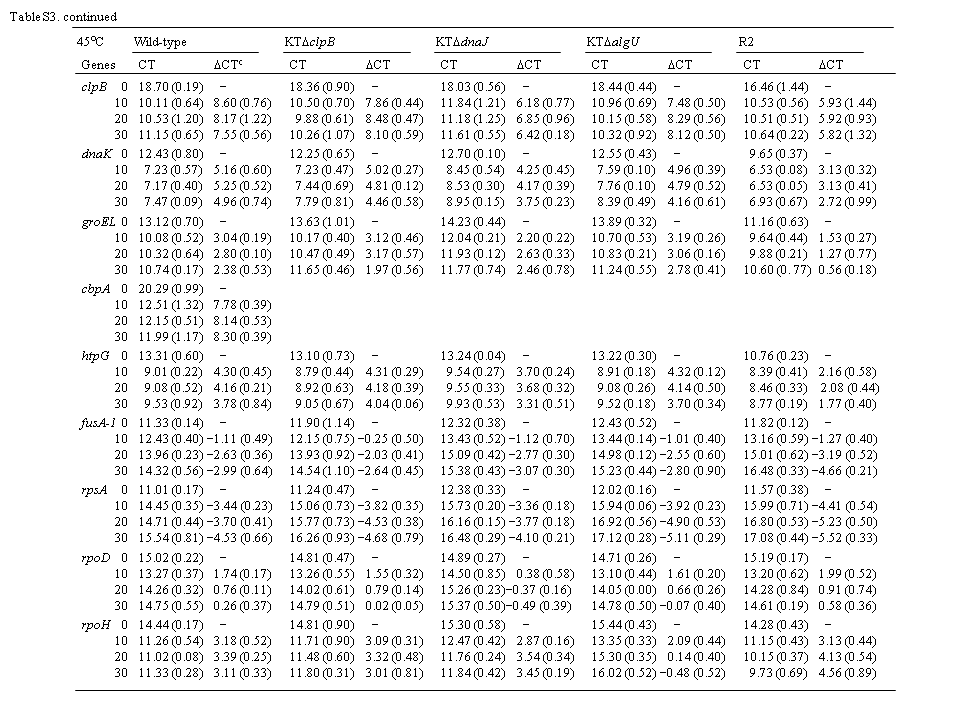

Supplement: Supplementary file 5 [file mbo30003-0922-sd5.tif]

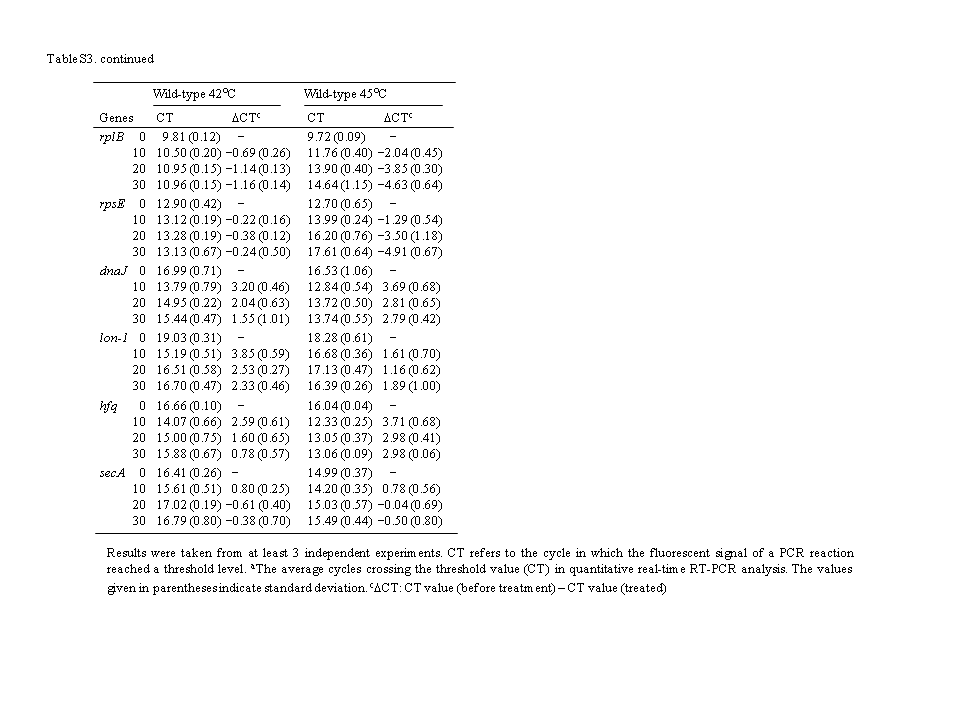

Supplement: Supplementary file 6 [file mbo30003-0922-sd6.tif]

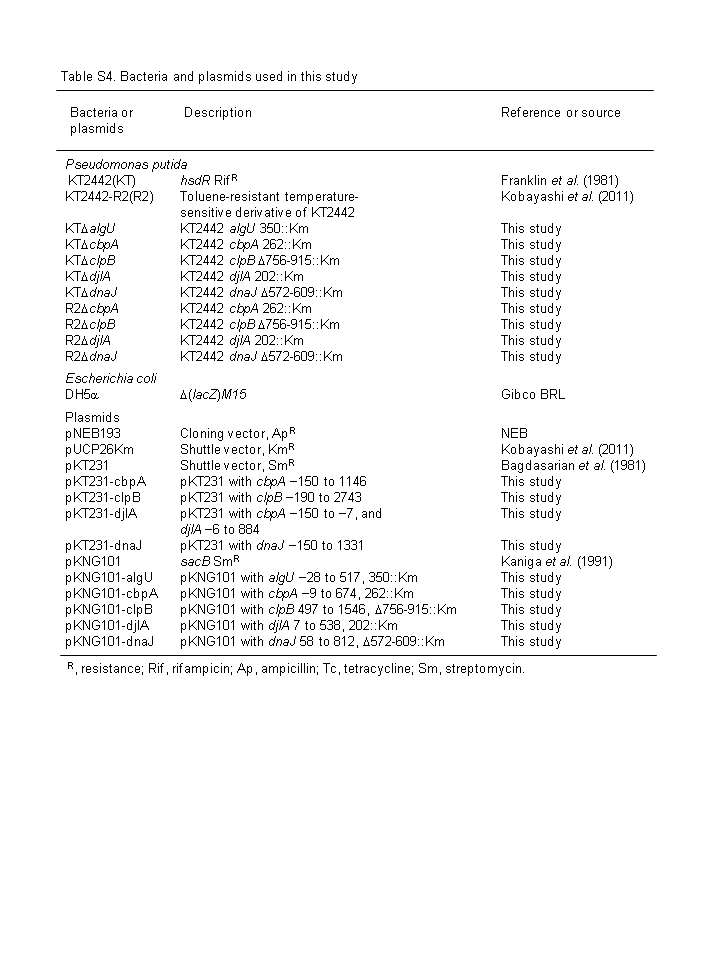

Supplement: Table S4 — Bacteria and plasmids used in the study. [file mbo30003-0922-sd7.tif]

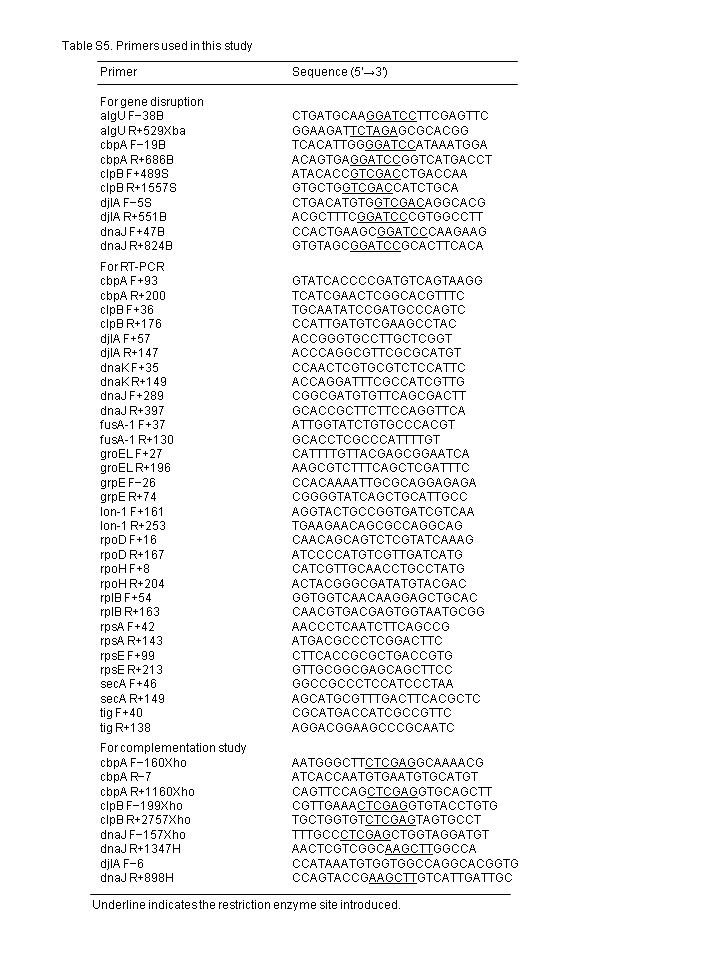

Supplement: Table S5 — Primers used in this study. [file mbo30003-0922-sd8.tif]
